# Connecting undergraduate and postgraduate medical education through an elective EPA-based transitional year in acute care: an early project report

**DOI:** 10.3205/zma001141

**Published:** 2017-11-15

**Authors:** Gersten Jonker, Reinier G. Hoff, Stefan Max, Cor J. Kalkman, Olle ten Cate

**Affiliations:** 1 University Utrecht, University Medical Center Utrecht, Department of Anaesthesiology, Utrecht, The Netherlands; 2 University Utrecht, University Medical Center Utrecht, Center for Research and Development in Medical Education, Utrecht, The Netherlands

**Keywords:** Undergraduate Medical Education, Graduate Medidal Education, Clinical Competence, Transitional year, Entrustable Professional Activities

## Abstract

**Objective: **A well-designed final year may ease the transition from medical school to postgraduate training, if it has enough depth to enable the acquisition of early specialty expertise, while keeping enough breadth to support the graduation as all-round physician. Aim of this article is to describe the design of a multidisciplinary dedicated transitional year (DTY) around the theme of recognition and initial treatment of vitally threatened patients.

**Methods: **Undergraduate and postgraduate training directors from the departments of Anaesthesiology, Cardiology, Emergency Medicine, Intensive Care Medicine and Respiratory Medicine at UMC Utrecht and partnering hospitals have collaboratively developed and implemented a curriculum for a final year focusing on three entrustable professional activities (EPAs) in the domain of acute care. These EPAs represent authentic tasks of starting residents in each of the participating specialties, align student training objectives with postgraduate expectations, and are the primary focus of learning, teaching, and assessment throughout the year. Students are developmentally supported by a mentor and educationally supported by monthly academic half days.

**Results:** Between October 2014 and November 2016,, 47 students chose DTY Acute Care. The set-up of our DTY is inspiring other specialties to develop multidisciplinary DTYs. Attainment of clinical competence, experience of students and staff, and exploration of graduates’ early careers are subjects of current research projects.

**Conclusion:** This multidisciplinary dedicated transitional year aims to graduate students with profile-specific competence in acute care. It prepares for residency in a range of specialties.

## Zusammenfassung

**Zielsetzung:** Ein gut gestaltetes letztes Studienjahr („praktisches Jahr” oder „Übergangsjahr“) kann den Übergang von Studium zu Facharztweiterbildung vereinfachen, wenn es ausreichend fachliche Tiefe zur frühen Aneignung von fachspezifischer Erfahrung bietet, ohne dabei die vollständige Bandbreite medizinischer Ausbildung zum praktischen Arzt zu vernachlässigen. Ziel dieses Artikels ist es, die Gestaltung eines multidisziplinären letzten Studienjahres („dedicated transitional year“, DTY) rund um die Erkennung und Erstbehandlung von vital bedrohten Patienten zu beschreiben.

**Methoden:** Die Lehrbeauftragten für Studenten- und Facharztausbildung der Anästhesiologie, Kardiologie, Notfallmedizin, Intensivmedizin und Pulmologie der Uniklinik Utrecht (Universitätsmedizinisches Zentrum, UMC) und seiner Lehrkrankenhäuser entwickelten und implementierten gemeinsam ein Curriculum für ein letztes Studienjahr, welches sich auf drei APT („anvertraubare professionelle Tätigkeit“, engl. „entrustable professional activity“, EPA) aus der Akutmedizin stützt. Diese APT repräsentieren tatsächliche Tätigkeiten beginnender Ärzte der teilnehmenden Fächer, gleichen die Lernziele der Studenten an diese zukünftigen Tätigkeiten an, und sind der Kern für Lernen, Lehre und Beurteilung während dieses Jahres. Die Studenten werden in ihrer Entwicklung durch einen Mentor unterstützt, und es findet monatlicher Unterricht statt.

**Ergebnisse: **Zwischen Oktober 2014 und November 2016 haben sich 47 Studenten für das DTY Akutmedizin entschieden. Die Einrichtung unseres DTY inspiriert andere Fachrichtungen, ebenfalls multidisziplinäre DTYs zu entwickeln. Die erlangte klinische Kompetenz, die Erfahrungen von Studenten und Mitarbeitern sowie die beginnenden Karrieren der Absolventen sind Gegenstand aktueller Forschungsprojekte.

**Fazit:** Dieses multidisziplinäre letzte Studienjahr soll Studenten ermöglichen, ihr Studium mit spezifischen Kompetenzen der Akutmedizin abzuschließen, und bereitet auf die Facharztweiterbildung in einer Reihe von Fachrichtungen vor.

## 1. Introduction

The goal of undergraduate medical training is to deliver doctors who are ready to practice within a postgraduate training programme [1]. 

Students, graduates and programme directors have identified gaps in the preparedness for postgraduate training in areas such as carrying responsibility and working at near intern-level, attributed to lack of educational focus in the final year of medical school [2], [3], [4]. A well-designed final year can bridge these gaps and facilitate the transition to residency [3], [5]. In the Netherlands, following the Bologna declaration [6], medical education consists of a three year Bachelor phase, which is mainly theoretical in nature, and a three year Master phase, which is largely devoted to clinical rotations [7]. Master years one and two comprise compulsory rotations in a wide range of disciplines. In the third and final year of the Master phase, all medical students at University Medical Center (UMC) Utrecht, follow a transitional year. This is a composite of one to three clinical electives and a research elective, which should ease the transition from undergraduate to postgraduate training. In this transitional year, students should work in clinical settings, integrate and apply knowledge and skills from previous years, grow into bearing clinical responsibility, deepen their understanding within areas of interest, acquire early specialty-specific expertise, and explore career options [2], [3], [4], [5].

Some students, who are determined about their choice of specialty, focus in an undergraduate transitional year on preparation for residency in one single specialty. It allows them to acquire early expertise, which may optimize their chances of getting into the desired residency programme. However, a transitional year aiming at one single discipline may not suit the numerous students who are undecided about their choice of specialty and wish to explore career options or want to work in more generalist fields. Moreover, during their final year, students initially convinced of their choice for a specialty may change their minds, or found to be ineligible or to lack aptitude. Additionally, a narrow, “overly dedicated” transitional year may not be the broad capstone of medical school that integrates knowledge and skills acquired in preceding years [3].

Purpose of this article is to describe the design of a final year of medical school shaped as a multidisciplinary, yet dedicated transitional year that addresses the above issues. Its goals are the acquisition of competence in acute care at an introductory level and to offer preparation for the responsibilities of postgraduate training in a range of specialties. We chose these goals as recently graduated doctors are often called upon to provide care in emergency situations.

## 2. Project description

### 2.1. A multidisciplinary thematic dedicated transitional year

The departments of Anaesthesiology, Cardiology, Emergency Medicine, Intensive Care Medicine and Respiratory Medicine at the UMC Utrecht, and St. Antonius Hospital, Nieuwegein, the Netherlands, have collaboratively developed a multidisciplinary thematic dedicated transitional year (DTY). The theme of our DTY is recognition and initial treatment of acutely compromised patients (therefore named DTY Acute Care). This is an important facet of all five participating disciplines, which combine an analytic approach with an interventional mind-set. At UMC Utrecht, students may elect to pursue our particular DTY. The twelve week research placement is chosen in one of the five involved specialties. The student chooses clinical rotations in three different specialties out of five partaking in the DTY. One placement is twelve weeks and two placements are six weeks each. This composition allows the student to gain concentrated but balanced clinical experience in areas of their interest, without being overly specialty-focused [5]. During the clinical rotations the students work at near-intern level to get accustomed to the roles and responsibilities of a resident [2], [3], [8]. This means that students get assigned identical tasks to those of junior residents, essential items are checked by supervisors, and prescriptions suggested by students need to be ratified by a physician.

Together with a six-week common Introductory block, holidays and a six-week Closing block, the transitional year completely covers the final year in medical school. 

#### 2.2. Learning objectives: Entrustable Professional Activities

##### 2.2.1. Entrustable Professional Activities

Because the DTY aims to facilitate the transition to residency, clinical tasks that starting residents must perform are suitable learning objectives [1], [9], [10]. Using tasks as learning objectives offers structure and clarity in the chaotic educational context of clinical practice [11]. It fosters learning by creating commitment and motivation [11]. Learning objectives, pertaining to clinical tasks, may be formulated as Entrustable Professional Activities [1], [9], [10]. An Entrustable Professional Activity (EPA) is a separately executable unit of clinical work, which can be performed with decreasing levels of supervision, and which may be entrusted to an individual that has been deliberately declared competent to execute that task without direct supervision [12]. The concept of EPAs fits well in the theoretical frameworks of Community of Practice and Situated Learning [13], which emphasise the importance of authentic tasks in (apprenticeship) learning. By being engaged in small but meaningful real world tasks, even novice learners may participate legitimately at the periphery of a community of practice and may progress towards the centre with growing experience [13]. The main learning objectives of DTY Acute Care are three EPAs that comprise relevant authentic tasks for starting residents covering “common ground” of the five participating specialties: “Recognition and initial treatment of patients with vital instability”, “Evaluation of patients with respiratory insufficiency”, and “Evaluation of patients with circulatory insufficiency” (see Attachment 1, Attachment 2 and Attachment 3). The three EPAs designate underpinning competencies, required knowledge, skills, and attitudes (cf [12]). The aim is to train students towards the level of performing these activities with indirect supervision (with post hoc review), which befits the level of responsibility that residents in their first year of training must take on [2], [10].

This set of three EPAs drives learning, teaching, and assessment in the entire DTY. The DTY EPAs were developed iteratively and collaboratively by experienced undergraduate and postgraduate faculty (n=13) of the involved departments. The developmental process took place between September 2014 and June 2015 and consisted of several meetings, separated and followed by iterative rounds of developing concept EPAs by email. Consensus was reached on the final product by all involved faculty members. The DTY EPAs are meant to align student training objectives with postgraduate expectations [10] and are special for not being confined to a single specialty, and thus transcend traditional barriers in being activities that are relevant for several specialties.

##### 2.2.2. Core EPAs versus DTY EPAs

During their rotations students are exposed to the breadth of the specialties they have chosen. In their work in Emergency Medicine, for example, they encounter patients with fractures, in Anaesthesiology they learn basics of providing anaesthesia, in Cardiology or Pulmonary Medicine they see patients with chronic disease. These aspects of their work are not part of the three DTY EPAs per se. The EPAs deal with the shared interest of the participating specialties in recognition and initial treatment of acutely compromised patients. However, work that is not confined to the EPAs is valuable in containing part-tasks that are useful experience for the EPAs (e.g. interpreting an ECG) and provides understanding of what the specialty encompasses.

The development of the DTY EPAs preceded the development of core EPAs for medical school at UMC Utrecht, a process that is currently underway. Core EPAs will provide scaffolding for learning of all students during the clinical phase, with the DTY EPAs providing advanced level objectives for the students choosing DTY Acute Care. The DTY EPAs intend to focus the attention of the student on these tasks during clinical placements that traditionally had no explicit learning objectives. So, the DTY EPAs can be seen as elective undergraduate EPAs [5], being more advanced than the core EPAs for entering residency that every medical graduate must master [10]. The EPAs can be linked to broader and more complex EPAs of postgraduate training in the participating specialties [9], [10], adding to the educational continuum in medical training. Currently, the EPA concept is being widely embraced in postgraduate medical training in the Netherlands [https://www.medischevervolgopleidingen.nl/epas]. 

#### 2.3. Assessment

EPAs and their assessment are used to focus experiential learning during the clinical placements. Students take a multimodal exam, blueprinted to the three EPAs, at the start and at the end of the DTY. This exam consists of several parts that test knowledge (written, closed and open format questioning), skills (several stations with Objective Structured Clinical Examinations), clinical reasoning (case-based discussions), and clinical performance in high-fidelity simulations of acute care settings. The pre-test focuses the students’ minds on the learning objectives and the expected performance level. The post-test, being similar to the pre-test, assesses the students personal development in competence.

Workplace-based assessment in our DTY is aligned with the EPAs. Short practice observations were developed collaboratively by the DTY team to cover observable units that are part of the EPAs (e.g. “Take a focused history of a dyspnoeic patient”, see Table 1 (Tab. 1)). These specific short practice observations are additional to the existing general ones in use in the master phase of medical school at UMC Utrecht and draw the attention of student and supervisor to the learning objectives of the DTY. Students document their practice observations in a paper-based portfolio, soon to be superseded by an electronic version.

Up until now, formal entrustment decisions on EPAs have not yet been part of summative evaluations of DTY students.

#### 2.4. Longitudinal support

The student is matched with one mentor for the entire year. The mentor is a senior resident in one of the participating specialties. This ‘buddy’ offers longitudinal developmental student support [14], serves as a role model, may assist in acculturation to the specialty, in career planning, in applying for residency, and in making most of the rotations [3], [4]. Mentors do not have a role in formal assessments of the mentee. Anecdotally, this mentorship is appreciated highly by both mentees and mentors.

Another special feature of the DTY is the monthly academic half-day. This is an interactive small group teaching session meant to exchange experiences of students and to discuss EPA-related topics from an integrative multidisciplinary viewpoint. In exit interviews, students report that it also fosters the motivating sense of belonging to a group, or class, whereas the usual transitional year in our medical school is purely individualistic. Our yearlong spaced-learning cycle of academic half-days covers the contents of intensive capstone bootcamp courses that are commonly employed in the transition to residency in the United States [5], [15]. 

## 3. Results

Between October 2014 and November 2016, we have enrolled 47 students in DTY Acute Care (about 7% of yearly cohorts). Until now, our multidisciplinary DTY Acute Care is unique in its sort. Other alliances of specialties at UMC Utrecht are currently teaming up to explore theme-oriented dedicated transitional years, following our example.

When entering the DTY, many of the students envision a career in one of the participating specialties. Ninety-two percent of students enrolling in the DTY primarily aim for a residency in one of the five specialties taking part in the DTY, with 16% of students being strongly determined of their choice. A minority wants to work in a discipline that does not participate in the DTY (8%). Of all students who are not fully decided in career choice at the start of their DTY, 68% consider specialties which do not take part in the DTY.

The DTY is open to all final year medical students at UMC Utrecht. Admission to the DTY is restricted by the limited availability of clinical placements. So far, we have been able to accommodate all interested students thanks to the flexibility in arranging the four placements and our regional collaborative partnership. There is no guarantee for a postgraduate residency placement or an accelerated residency programme when taking part in the DTY. 

The DTY is subject of three ongoing evaluative research studies. One study evaluates the efficacy of the DTY to develop clinical competence in the three multidisciplinary EPAs. A second study explores the experience of students and staff with the DTY. A third study is a follow-up of graduated students from DTY exploring their early careers. 

## 4. Discussion

We designed DTY Acute Care around the theme of evaluation and initial management of vitally compromised patients. We have set clearly articulated multidisciplinary learning objectives that are authentic tasks of entering residents. This orientation on a theme, important for future doctors in many specialties, is one of its strengths. It assures enough breadth to graduate as a generalist, whilst offering enough depth to gain useful competence for postgraduate specialty training. Moreover, the theme-orientation and the learning objectives provide coherence in a year consisting of elective rotations.

The design and implementation of the DTY has been a labour intensive, albeit satisfying, process. It took considerable time and effort to collaboratively develop and describe the three EPAs, transcending traditional specialty boundaries, with faculty from five disciplines. We feel that it did reduce thinking in silos in the specialties. 

The EPAs are the focus of assessment. The multimodal exam at the start and end of the DTY may be an important way to demonstrate attained competence. However, it is labour intensive as well and may not be a sustainable undertaking at larger scale. Workplace assessment has been linked to the EPAs by using the themed short clinical observation forms. In 2014, the DTY adopted an EPA approach in a medical school curriculum that was not EPA-based at the time. Therefore, entrustment decisions have not yet been part of summative evaluations of DTY students. An entrustment decision relies on multiple observations of clinical performance and takes test results of required knowledge and skills into account. After an entrustment decision the student is permitted to execute the activity with indirect supervision. With the advent of our new EPA-based medical school curriculum, entrustment decisions will inevitably become part of evaluating students in the clinical phase, including the DTY. The attainment of EPAs, even at the undergraduate level of near-independence, may help to justify decisions to delegate tasks or to reconsider the appropriateness of delegation to entering residents in the light of patient safety [10], [16].

DTY students do their rotations in the five specialties in several partnering regional teaching hospitals around the region. Uptake of the DTY concept and principles will likely not be homogenous. In addition, several kinds of clinical students may do rotations in hospitals –even at the same time. It is therefore the principal responsibility of students themselves to continuously draw the attention of local supervisors to the DTY and its special objectives. At the same time, investments should be made to adequately prepare supervisors and mentors and inform them on a continuing basis.

## 5. Conclusion

In this article we describe the development and design of a multidisciplinary dedicated transitional year, around the theme of acute care, with entrustable professional activities as learning objectives. After the DTY, students have learned to bear responsibility for patients, have refreshed, enhanced, and integrated knowledge and skills from previous years, and will have acquired a profile of competence in acute care at the level of near-independent performance; this should be useful preparation for residency in the participating specialties. A similar set-up could be used to design other multidisciplinary dedicated transitional years either organised around related disciplines or around various themes (profiles) in medicine. This would enable more students to graduate with profile-specific competence, well prepared to enter residency in a range of specialties.

## Competing interests

The authors declare that they have no competing interests. 

## Supplementary Material

Elaborated entrustable professional activity on recognition and initial treatment of vitally threatened patients

Elaborated entrustable professional activity on evaluation of respiratory compromised patients

Elaborated entrustable professional activity on evaluation of circulatory compromised patients

## Figures and Tables

**Table 1:**
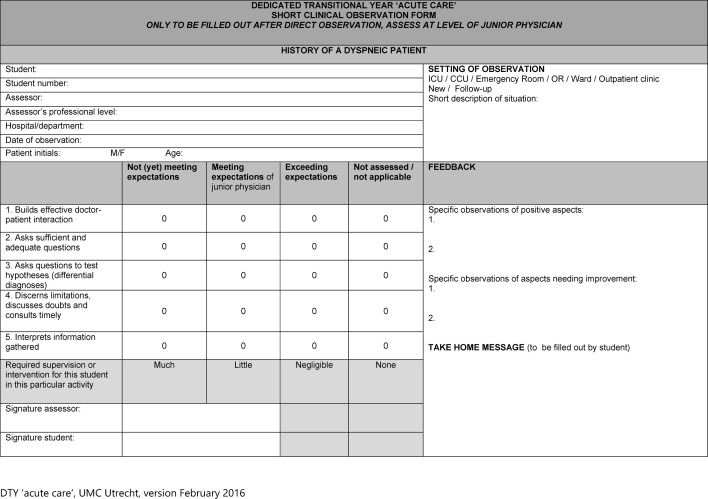
Example of a short clinical observation form to link workplace assessment to the DTY EPAs.
