# Supplementary material for: Connecting undergraduate and postgraduate medical education through an elective EPA-based transitional year in acute care: an early project report
Source: GMS J Med Educ. 2017 Nov 15;34(5):Doc64. doi: 10.3205/zma001141 (PMC5704612; doi:10.3205/zma001141)
Supplement: Elaborated entrustable professional activity on recognition and initial treatment of vitally threatened patients [file JME-34-64-s-001.pdf]

**Title**	Recognition and initial treatment of patients with vital instability
**Description**	• Evaluate patient with the 'ABCD' approach;<br>• Measure and interpret vital signs;<br>• Based on the above, come to a clinical evaluation;<br>• In case of emergency: call directly for help/supervision;<br>• Initiate Basic Life Support, perform bag-mask-ventilation and airway manoeuvres and start intravenous fluid therapy;<br>• Be a member of the resuscitation team;<br>• Discern limitations in capability.<br><br>This EPA does <u>not</u> comprise:<br>• Provision of advanced cardiac/trauma life support, completely and/or indirectly supervised<br>• Care for paediatric patients with vital instability (< 16 years)
**Link to competency domains**	CanMEDS (Dutch KNMG version):<br>• Medical Expert: applies diagnostic, therapeutic, preventive repertoire (1.2)<br>• Communicator: reports adequately on a case, orally and in writing (2.4)<br>• Collaborator: consults others effectively (3.1), efficacious in interdisciplinary collaboration (3.4)<br>• Manager: works effectively within a system (6.2)<br>• Professional: discerns limits of competence (7.3)
**Required knowledge, skills, and attitudes (level of doctor in postgraduate year 1)**	<u>*Knowledge*</u><br>• Knowledge of normal ranges for blood pressure, heart rate, respiratory rate, saturation and temperature;<br>• Knowledge of symptoms of threatened vital parameters<br>• Knowledge of Glasgow Coma Scale (GCS) and primary neurological survey;<br>• Knowledge of causes of disturbances in consciousness;<br>• Knowledge of Basic Life Support;<br>• Knowledge of principles of advanced cardiac and trauma life support;<br>• Knowledge of 4Hs, 4Ts, shock and no-shock algorithms;<br>• Knowledge of indications for and application of (Automatic) External Defibrillators;<br>• Knowledge of effective communication protocols to call for help (e.g. SBARR method);<br>• Knowledge of local protocols regarding acute intervention teams, emergency telephone number, resuscitation team etcetera;<br>• Knowledge of equipment and materials for basic interventions in 'ABC' (e.g. infusion materials, AMBU-bag, non-rebreathing mask, Mayo tube) including (contra-)indications;<br>• Knowledge of levels of care on different wards (normal ward to ICU);<br>• Knowledge of indications to stop resuscitation efforts<br><br><u>*Skills*</u><br>• Asks timely for help/supervision with an effective communication protocol (e.g. SBARR) and locally applicable emergency procedure;<br>• Performs and interprets a systematic physical exam according to 'ABCD' and monitoring of vital signs;

	• Clinical judgment based on 'ABCD' and vital parameters;<br>• Provides probable diagnosis regarding the cause of the vital instability;<br>• Gives and receives orders/tasks and confirms the order/task and its execution;<br>• Proposes a management plan;<br>• Proposes orders regarding treatment;<br>• Initiates Basic Life Support;<br>• Performs Basic Life Support skills and part-tasks;<br>• Performs bag-mask ventilation and airway manoeuvres (head tilt/chin lift, jaw thrust);<br>• Sites a peripheral intravenous catheter;<br>• Defibrillates a shockable rhythm with (automatic) external defibrillator;<br>• Reports in medical record;<br>• Provides a structured handover of a patient with vital instability to colleague/supervisor/ward.<br><br>*Attitudes*<br>• Discerns and acknowledges personal limits of knowledge, skill and capability and can adequately reflect on this;<br>• Situational awareness;<br>• Is set to short cycles of assessment, treatment, and re-assessment;<br>• Collaborates in a team in an emergency setting;<br>• Reveals professional role and level;<br>• Professional conduct towards patient and/or relatives;<br>• Uses Evidence Based Medicine.
**Information to assess progress**	*Workplace assessment*<br>• Mini-CEXs with regard to the evaluation of patients with or without vital instability, including indicating necessity for (acute) intervention and with regard to discernment of personal limits of capability;<br>• Multisource feedback: performance as a team member in urgent and non-urgent settings;<br><br>*Assessment of knowledge, skills and attitudes*<br>• Knowledge examination (written, variety of formats);<br>• Clinical reasoning and know-how (case-based discussions);<br>• Demonstration of isolated skills in non-clinical setting (Objective Structured Examination of Clinical Skills);<br>• Reflection forms regarding performance, difficult moments, discernment of limitations.<br><br>*Assessment of clinical performance*<br>• Simulation of acute care settings.
**Target level of supervision (entrustment)**	Indirect supervision (immediately available): evaluation and initial management, awaiting arrival of help/supervisor
**When is unsupervised practice expected?**	At the end of DTY Acute Care
