# Supplementary material for: Connecting undergraduate and postgraduate medical education through an elective EPA-based transitional year in acute care: an early project report
Source: GMS J Med Educ. 2017 Nov 15;34(5):Doc64. doi: 10.3205/zma001141 (PMC5704612; doi:10.3205/zma001141)
Supplement: Elaborated entrustable professional activity on evaluation of respiratory compromised patients [file JME-34-64-s-002.pdf]

**Title**	Evaluation of patients with respiratory insufficiency
**Description**	• Rapid evaluation with the 'ABCD' approach;<br>• Take a focused history of a patient with respiratory insufficiency in an Emergency Room, Coronary Care Unit, ward or urgent outpatient clinic (outside Operating Room and Intensive Care settings) ;<br>• Perform a physical exam, including 'ABCD' and vital signs;<br>• Order and interpret *basic* diagnostic tests (blood, chest X-ray, ECG);<br>• Draft and rank a differential diagnosis in a patient with respiratory insufficiency;<br>• Propose initial management and treatment plans.<br><br>This EPA does <u>not</u> comprise:<br>• Care for the patient (nearly) in a cardiopulmonary resuscitation setting<br>• Care for paediatric patients with respiratory instability (< 16 years)<br>• Interpretation of advanced diagnostic tests (Ventilation-Perfusion scans, spirometry)
**Link to competency domains**	CanMEDS (Dutch KNMG version):<br>• Medical Expert: applies diagnostic, therapeutic, preventive repertoire (1.2)<br>• Communicator: creates effective therapeutic relationships (2.1), reports adequately on a case, orally and in writing (2.4)<br>• Collaborator: consults others effectively (3.1)<br>• Scholar: appraises medical information critically (4.1)<br>• Health Advocate: recognises determinants of disease (5.1)<br>• Manager: works effectively within a system (6.2)<br>• Professional: adequate (inter-)professional conduct (7.2), discerns limits of competence (7.3)
**Required knowledge, skills, and attitudes (level of doctor in postgraduate year 1)**	*<u>Knowledge</u>*<br>• Knowledge of normal ranges for blood pressure, heart rate, respiratory rate, saturation and temperature;<br>• Knowledge of relevant criteria and scores (e.g. SIRS-criteria, Wells score; GOLD-criteria; Pneumonia Severity Index; Pneumonia Likelihood Ratio; AMBU-65 and CURB-65; ALI/ARDS);<br>• Knowledge of criteria for ventilation and admission to ward or ICU;<br>• Knowledge of (results of) relevant diagnostic tests (i.e. laboratory blood tests, blood gas analysis, chest X-ray and systematic interpretation, ECG);<br>• Knowledge of results of pleural fluid investigations (chemistry, microbiology);<br>• Knowledge of most important disorders leading to respiratory insufficiency;<br>• Knowledge of options in additional diagnostic testing (e.g. rapid PCR tests, viral, bacterial and fungal tests, CT scan, VP scan);

<table>
<tr><td></td><td>

Knowledge of indications for ventilation;
Knowledge of indications and techniques of oxygen therapy (F_iO_2, nasal cannulae, non-rebreathing mask, CPAP, Venturi, Optiflow);
Knowledge of indications for chest drains;
Knowledge of indications for bronchoscopy;
Knowledge of initial pharmacological treatment of important disorders leading to respiratory insufficiency.

<u>Skills</u>

Performs and interprets a systematic physical exam according to ‘ABCD’ and monitoring of vital signs;
Asks timely for help/supervision;
Takes a focused history and performs a focused physical exam in patients with respiratory insufficiency;
Performs a blood gas analysis;
Interprets result of a blood gas analysis;
Connects patient to monitoring such as ECG/telemetry, non-invasive blood pressure, pulse oximetry, and interprets findings;
Interprets 12-lead ECG: myocardial ischemia, rhythm- and conduction disorders;
Recognises myocardial ischemia, rhythm- and conduction disorders on telemetry monitor;
Writes an order for chest X-ray;
Systematic interpretation of chest X-ray and recognition of relevant radiologic findings;
Interprets findings of pleural fluid tests;
Sites a peripheral intravenous catheter;
Drafts and ranks a differential diagnosis and provides a probable diagnosis in a patient with circulatory insufficiency;
Proposes a management plan;
Proposes orders regarding treatment;
Reports in medical record and provides a structured handover.

<u>Attitudes</u>

Discerns and acknowledges personal limits of knowledge, skill and capability and can adequately reflect on this;
Is set to short cycles of assessment, treatment, and re-assessment;
Reveals professional role and level;
Collaborates with ward staff;
Professional conduct towards patient and/or relatives;
Uses Evidence Based Medicine.

</td></tr>
<tr><td>Information to assess progress</td><td>
<u>Workplace assessment</u>

Mini-CEXs with regard to the evaluation of patients with or without vital instability, including indicating necessity for (acute) intervention and with regard to discernment of personal limits of capability;
Multisource feedback: performance as a team member in urgent and non-urgent settings;

</td></tr>
</table>

	Assessment of knowledge, skills and attitudes<br>• Knowledge examination (written, variety of formats);<br>• Clinical reasoning and know-how (case-based discussions);<br>• Demonstration of isolated skills in non-clinical setting (Objective structured examination of skills);<br>• Reflection forms regarding performance, difficult moments, discernment of limitations.<br><br>*Assessment of clinical performance*<br>• Simulation of acute care settings.
**Target level of supervision (entrustment)**	Indirect supervision (immediately available): evaluation and initial management, awaiting arrival of help/supervisor
**When is unsupervised practice expected?**	At the end of DTY Acute Care
