# Supplementary material for: Connecting undergraduate and postgraduate medical education through an elective EPA-based transitional year in acute care: an early project report
Source: GMS J Med Educ. 2017 Nov 15;34(5):Doc64. doi: 10.3205/zma001141 (PMC5704612; doi:10.3205/zma001141)
Supplement: Elaborated entrustable professional activity on evaluation of circulatory compromised patients [file JME-34-64-s-003.pdf]

<table>
<tr><td>Title</td><td>Evaluation of patients with circulatory insufficiency</td></tr>
<tr><td>Description</td><td>

Rapid evaluation with the ‘ABCD’ approach;
Take a focused history of a patient with circulatory insufficiency in an Emergency Room, Coronary Care Unit, ward or urgent outpatient clinic (outside Operating Room and Intensive Care settings) ;
Perform a physical exam, including ‘ABCD’ and vital signs;
Order and interpret basic diagnostic tests (blood, chest X-ray, ECG);
Draft and rank a differential diagnosis in a patient with circulatory insufficiency;
Propose initial management and treatment plans.

This EPA does <u>not</u> comprise:

Care for the patient (nearly) in a cardiopulmonary resuscitation setting
Care for paediatric patients with circulatory instability (< 16 years)
Interpretation of advanced diagnostic tests (cardiac catheterisation, echocardiography)

</td></tr>
<tr><td>Link to competency domains</td><td>
CanMEDS (Dutch KNMG version):

Medical Expert: applies diagnostic, therapeutic, preventive repertoire (1.2)
Communicator: creates effective therapeutic relationships (2.1), reports adequately on a case, orally and in writing (2.4)
Collaborator: consults others effectively (3.1)
Scholar: appraises medical information critically (4.1)
Health Advocate: recognises determinants of disease (5.1)
Manager: works effectively within a system (6.2)
Professional: adequate (inter-)professional conduct (7.2), discerns limits of competence (7.3)

</td></tr>
<tr><td>Required knowledge, skills, and attitudes (level of doctor in postgraduate year 1)</td><td>
<u>Knowledge</u>

Knowledge of normal ranges for blood pressure, heart rate, respiratory rate, saturation and temperature;
Knowledge of relevant criteria and scores (e.g. Crusade, Heartscore, TIMI, CHADS2VASC, SIRS-criteria, hypovolemic shock classification, NYHA classification);
Knowledge of different types of shock and their treatments;
Knowledge of levels of care on different wards (normal ward to ICU);
Knowledge of (results of) relevant diagnostic tests (i.e. laboratory blood tests, blood gas analysis, chest X-ray and systematic interpretation, ECG);
Knowledge of intravenous fluid therapy protocols;
Knowledge of most important disorders leading to circulatory insufficiency (i.e. different types of shock);
Knowledge of indications for echocardiography and of relevant reported findings;
Knowledge of indications for cardiac catheterisation (and percutaneous cardiac intervention) and of relevant reported findings;
Knowledge of initial (pharmacological) treatment of different types of shock. Inotropes and vasoactive medications.

</td></tr>
</table>

	Skills<br>• Performs and interprets a systematic physical exam according to ‘ABCD’ and monitoring of vital signs;<br>• Asks timely for help/supervision;<br>• Takes a focused history and performs a focused physical exam in patients with circulatory insufficiency;<br>• Performs a blood gas analysis;<br>• Interprets result of a blood gas analysis;<br>• Connects patient to monitoring such as ECG/telemetry, non-invasive blood pressure, pulse oximetry, and interprets findings;<br>• Evaluates heart rhythm on telemetry monitor;<br>• Interprets 12-lead ECG : myocardial ischemia, rhythm- and conduction disorders;<br>• Writes an order for chest X-ray;<br>• Systematic interpretation of chest X-ray and recognition of relevant radiologic findings;<br>• Sites a peripheral intravenous catheter;<br>• Drafts and ranks a differential diagnosis and provides a probable diagnosis in a patient with circulatory insufficiency;<br>• Proposes a management plan;<br>• Proposes orders regarding treatment;<br>• Reports in medical record and provides a structured handover.<br><br>*Attitudes*<br>• Discerns and acknowledges personal limits of knowledge, skill and capability and can adequately reflect on this;<br>• Is set to short cycles of assessment, treatment, and re-assessment;<br>• Reveals professional role and level;<br>• Collaborates with ward staff;<br>• Professional conduct towards patient and/or relatives;<br>• Uses Evidence Based Medicine.
**Information to assess progress**	*Workplace assessment*<br>• Mini-CEXs with regard to the evaluation of patients with or without vital instability, including indicating necessity for (acute) intervention and with regard to discernment of personal limits of capability;<br>• Multisource feedback: performance as a team member in urgent and non-urgent settings;<br>*Assessment of knowledge, skills and attitudes*<br>• Knowledge examination (written, variety of formats);<br>• Clinical reasoning and know-how (case-based discussions);<br>• Demonstration of isolated skills in non-clinical setting (Objective Structured Examination of Clinical Skills);<br>• Reflection forms regarding performance, difficult moments, discernment of limitations.<br>*Assessment of clinical performance*<br>• Simulation of acute care settings.
**Target level of supervision (entrustment)**	Indirect supervision (immediately available): evaluation and initial management, awaiting arrival of help/supervisor
**When is unsupervised practice expected?**	At the end of DTY Acute Care
